# Editorial: Stigma's Impact on People With Mental Illness: Advances in Understanding, Management, and Prevention

**DOI:** 10.3389/fpsyg.2021.715247

**Published:** 2021-07-09

**Authors:** Alexandre Andrade Loch, Alexandre Paim Diaz, Antonio Pacheco-Palha, Milton L. Wainberg, Antonio Geraldo da Silva, Leandro Fernandes Malloy-Diniz

**Affiliations:** ^1^Institute of Psychiatry, University of São Paulo, São Paulo, Brazil; ^2^Instituto Nacional de Biomarcadores em Neuropsiquiatria (INBioN), Conselho Nacional de Desenvolvimento Científico e Tecnológico, São Paulo, Brazil; ^3^Louis A. Faillace, MD, Department of Psychiatry and Behavioral Sciences, The University of Texas Health Science Center at Houston, Houston, TX, United States; ^4^Faculdade de Medicina, Universidade Do Porto, Porto, Portugal; ^5^Department of Psychiatry, Columbia University, New York, NY, United States; ^6^Brazilian Psychiatry Association - Associação Brasileira de Psiquiatria (ABP), Rio de Janeiro, Brazil; ^7^Federal University of Minas Gerais, Belo Horizonte, Brazil

**Keywords:** stereotype (psychology), prejudice & discrimination, mental health professional, empowerment, hysteria

The stigma toward mental illness is a persistent problem. Stemming from our tribal necessity to separate “them” from “us” to increase our belonging to a certain group, stigma endures and transmutes itself across time. From the witch hunts in the XVI and XVII centuries to the vanishing of the term hysteria from diagnostic manuals, the prejudice related to mental illness' stigma assume diverse forms (Loch and Wang, 2012). Increasing efforts have been carried out to understand and reduce it, reflected in an escalating number of works published on the issue in the international literature in the past 20 years. Nevertheless, still in the present days people with mental disorders continue to suffer with distorted opinions and prejudiced attitudes coming from a multitude of sources. For example, patients netative attitudes directed to own's mental illness (self-stigma) figures as an frequent cause of avoidance to help-seeking (Loch et al., 2013). Other one of these sources of stigma are mental health professionals (Schnyder et al., 2017; Valery and Prouteau, 2020). These gatekeepers should be addressed regarding prejudiced opinions, for they could act as important barriers preventing people with mental illness from seeking adequate help for their general health issues (Clement et al., 2015). In this sense, Wu et al. examined stigmatizing attitudes in non-mental health professionals in several Chinese hospitals. Authors found that most professionals held prejudiced opinions toward people with mental illness. Their beliefs were informed predominantly by mass-media information—one of the greatest sources of misinformation, responsible for perpetuating stereotypes related to psychiatric disorders. This was also observed in non-psychiatry doctors and in medical students in Portugal in the study conducted by Oliveira et al. This reinforces the idea that the struggle between “insiders” and “outsiders” is still taking place. And the label of “mentally ill” —evoking the idea of pathological behavioral changes and “madness” in people's common belief—would constitute a vulnerability to an outsider status. The insider vs. outsider segregation is particularly depicted by Martinez's et al. interesting results, showing that immigrant status was the only variable significantly related to personal depression stigma in adolescents in Chile and Colombia.

These results update and integrate a large corpus of research that nourishes puissant gold-standart tools to fight stigma: information, contact and protest (Corrigan and Penn, 1999). Information to dismantle biased and distorted public beliefs about mental illness. Contact—with individuals with psychiatric disorders—should further demystify negative stereotypes attached to those with mental disorders. And protest/empowerment should make people with the disorders deny currently circulating stereotypes and make them fight for their rights—fight public and structural stigma seen in mental healthcare delivery, for instance. However, it seems that stigma resists even to high-profile national campaigns employing these powerful tools. Walsh and Foster address this issue in depth in their work “Why do the public resist efforts to challenge mental health related stigma? A critical review of public health campaigns.” Authors discuss the social processes which “Other” individuals with experiences of mental illness, taking a closer look into these campaigns and suggesting possible subtle mechanisms that might be undermining their effectiveness.

At last, we bring then to the reader of our Research Topic works on promising interventions delivering diverse ways to fight stigma. One of the greatest issues feeding self-stigma and causing stress for those with psychiatric disorders is the diagnosis' secrecy. In this sense, Modelli et al. present an interesting adaptation of the Honest-Open-Proud (HOP) protocol to the Brazilian context. The HOP-based program addressed the stigma and stress related to disclosing or not one's diagnosis of mood disorder and was accessed through a controlled trial. Depressed individuals under the intervention group improved in their perception of stigma as a stress, and depressed and bipolar individuals improved in their feelings of authenticity. As to accurate information to reduce stigma, Ueda et al. conducted an educational program with schoolteachers consisting in a 50-min video lesson designed to improve mental health literacy. It was efficacious in improving knowledge in mental disorders, and improved teachers' intention to assist students with depression. Surprisingly, it was not successful in decreasing the stigma toward mental illness *per se*. Likewise, Tan et al. showed the efficacy of a knowledge-contact-based intervention in improving university students' stigma. 309 students had to attend an one-off intervention which comprised a lecture on depression and personal contact with a person with lived experience of mental illness. After the intervention, their recognition of depression and help-seeking preferences improved, suggesting this brief tool as an important one to tackle stigma as a barrier to treatment. Focusing on the main stakeholders to understand stigmatization mechanisms and individuals' empowerment, Ong et al. employed focus group discussions with 42 individuals with mental illness to analyze their experience with stigma. Public and structural stigma were the main themes that emerged in participant's everyday life. However, 4 themes regarding participant's strategy do reduce stigma were also identified: non-disclosure of condition, standing up for themselves, individual efforts in raising awareness, and improving themselves and live life as per normal. The three last ones are prototypical of what should be employed by people with mental disorders: stereotype disconfirmation, empowerment and protest.

By bringing this state-of-the art Research Topic, we wish to inform the reader about the several aspects of stigma. Our goal is to provide insights so that the reader can echo our concern and embrace our cause in reducing the suffering of people with psychiatric disorders. Fighting the stigma of mental illness is like catching a fish with one's own hands: it's tricky, slippery. One needs to employ the right tools and have the right information to do so in a way that effectively works.

## Author Contributions

All authors listed have made a substantial, direct and intellectual contribution to the work, and approved it for publication.

## Conflict of Interest

The authors declare that the research was conducted in the absence of any commercial or financial relationships that could be construed as a potential conflict of interest.
